# Investigation of Nanoparticle Metallic Core Antibacterial Activity: Gold and Silver Nanoparticles against *Escherichia coli* and *Staphylococcus aureus*

**DOI:** 10.3390/ijms22041905

**Published:** 2021-02-14

**Authors:** Jimmy Gouyau, Raphaël E. Duval, Ariane Boudier, Emmanuel Lamouroux

**Affiliations:** 1 Université de Lorraine, CNRS, L2CM, F-54000 Nancy, France; gouyau.jimmy@gmail.com; 2 ABC Platform^®^, F-54505 Vandœuvre-lès-Nancy, France; 3 Université de Lorraine, CITHEFOR, F-54000 Nancy, France; Ariane.Boudier@univ-lorraine.fr

**Keywords:** metallic nanoparticles, silver, gold, synthesis, characterization, surface reactivity, stability, antibacterial activity, *Escherichia coli*, *Staphylococcus aureus*, multidrug resistant bacteria

## Abstract

Multidrug-resistant (MDR) bacteria constitute a global health issue. Over the past ten years, interest in nanoparticles, particularly metallic ones, has grown as potential antibacterial candidates. However, as there is no consensus about the procedure to characterize the metallic nanoparticles (MNPs; i.e., metallic aggregates) and evaluate their antibacterial activity, it is impossible to conclude about their real effectiveness as a new antibacterial agent. To give part of the answer to this question, 12 nm gold and silver nanoparticles have been prepared by a chemical approach. After their characterization by transmission electronic microscopy (TEM), Dynamic Light Scattering (DLS), and UltraViolet-visible (UV-vis) spectroscopy, their surface accessibility was tested through the catalytic reduction of the 4-nitrophenol, and their stability in bacterial culture medium was studied. Finally, the antibacterial activities of 12 nm gold and silver nanoparticles facing *Staphylococcus aureus* and *Escherichia coli* have been evaluated using the broth microdilution method. The results show that gold nanoparticles have a weak antibacterial activity (i.e., slight inhibition of bacterial growth) against the two bacteria tested. In contrast, silver nanoparticles have no activity on *S. aureus* but demonstrate a high antibacterial activity against *Escherichia coli*, with a minimum inhibitory concentration of 128 µmol/L. This high antibacterial activity is also maintained against two MDR-*E. coli* strains.

## 1. Introduction

Antimicrobial resistance (AMR) is a vital public health issue [1]. In particular, antibiotic resistance and the spread of multidrug-resistant (MDR) bacteria are a global issue [2]. If nothing is done to combat AMR, the most pessimistic projections predict 10 million deaths per year by 2050 [3]. A recent Centers-for-Disease-Control-and-Prevention (CDC) report shows that more than 2.8 million antibiotic-resistant infections occur each year in the United States, and more than 35,000 people die [4]. In the same way, MDR-bacteria are also responsible for 33,000 deaths by year, in Europe [5]. Among these bacteria, Gram-positive bacteria, like *Staphylococcus aureus*, and Gram-negative ones, such as Enterobacteriaceae, *Pseudomonas aeruginosa*, and *Acinetobacter baumannii*, are the most frequently encountered bacteria in human infections [6]. Developing new and effective antibiotics against these bacteria is therefore a high priority emergency [7].

An exciting approach to address this issue can be metallic nanoparticles (MNPs) as new antibacterial agents. Indeed, metallic (e.g., Ag, Au, Cu…) nanoparticles’ antibacterial activity facing numerous bacteria has been reported [8,9,10,11,12,13]. Interestingly, the concerned studies deal mainly with silver or gold nanoparticles. Among these, most studies are interested in NPs associated with antibiotics or any other potentially active molecules, such as chitosan; even if it is difficult to know what the antibacterial activity relates to, they attributed the activity to the MNPs. However, by definition, an MNP is an aggregate of metallic atoms with a specific size ranging from 1 to 100 nm [14]. To control their size and size distribution, it is necessary to stabilize their surface using surfactants. Consequently, the metallic aggregate (metallic core) and the stabilizers (organic shell) are, most of the time included in the term MNPs, unconsciously. Moreover, many studies do mention neither the origin of bacteria nor the corresponding antibiograms for a clinical isolate. All of this raises the question of the real effectiveness of MNPs (i.e., metallic aggregates) as a new antibacterial agent. To provide some elements of answer to this question, it appears necessary to have an adequate and complete description of both tested nano-objects and bacteria, allowing to attribute evaluation results to MNPs [15].

Hence, we have synthesized and characterized gold or silver nanoparticles and then determined their antibacterial activities. Silver nanoparticles were synthesized from aqueous silver nitrate, sodium tricitrate, and NaBH_4_, whereas gold nanoparticles were prepared using the Turkevich procedure [16]. The full characterization of these nanoparticles performed by UV-visible spectroscopy, transmission electron microscopy (TEM), and dynamic light scattering (DLS) is detailed. The analyses indicate that the synthesis procedures are repeatable for the preparation of 12 nm spherical citrate-capped nanoparticles, exhibiting a narrow size distribution. The antibacterial activity of both nanoparticles was evaluated, using the broth microdilution method, against *Staphylococcus aureus* (ATCC 29213), *Escherichia coli* (ATCC 25922), and two antibiotic-resistant *E. coli* strains: EcR1 (penicillinase) and EcR2 (cephalosporinase overproduction).

## 2. Results

### 2.1. Characterization of Citrate-Capped Gold and Silver Nanoparticles

Citrate-capped gold nanoparticles (AuNPs) have been prepared following the Turkevich approach. Briefly, an aqueous solution of trisodium citrate is added to a refluxed aqueous solution of HAuCl_4_. The color of the HAuCl_4_ solution immediately changes from yellow to black before becoming red-wine. For citrate-capped silver nanoparticles (AgNPs), sodium borohydride and citrate have been used as reductant and stabilizer, respectively. They are added as an aqueous solution to AgNO_3_ at room temperature. The resulting solution turns dark brown and then lightens. At least three independent experiments of these two kinds of nanoparticles have been prepared and characterized.

#### 2.1.1. Transmission Electronic Microscopy

**Figure 1:**
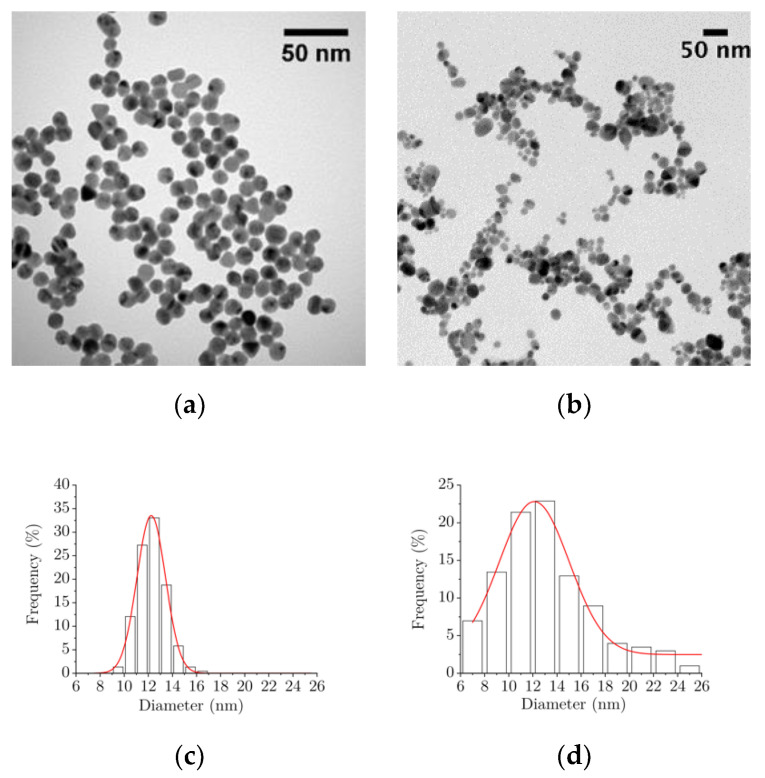
TEM micrographs and size distributions of (**a**,**c**) gold nanoparticles (AuNPs) and (**b**,**d**) silver nanoparticles (AgNPs).

The detailed morphology of the citrate-capped gold and silver nanoparticles has been characterized by TEM. Figure 1 shows representative micrographs and statistical analysis of these samples. Both kinds of samples appear as spheroidal nanoparticles (Figure 1a,b). Few anisotropic shapes such as triangles are observed in the AuNPs sample, mainly constituted of spherical nanoparticles of similar size (Figure 1a). In contrast, the AgNPs sample exhibits spherical nanoparticles of different sizes (Figure 1b). Statistical analysis of the size distribution of the AuNPs and AgNPs highlights that both have a mean diameter close to 12 nm. Moreover, the AuNPs and AgNPs populations follow a Gaussian distribution (Figure 1c,d): 12.1 nm, σ = 1.1, and 12.2 nm, σ = 2.7, respectively.

#### 2.1.2. Dynamic Light Scattering

**Figure 2:**
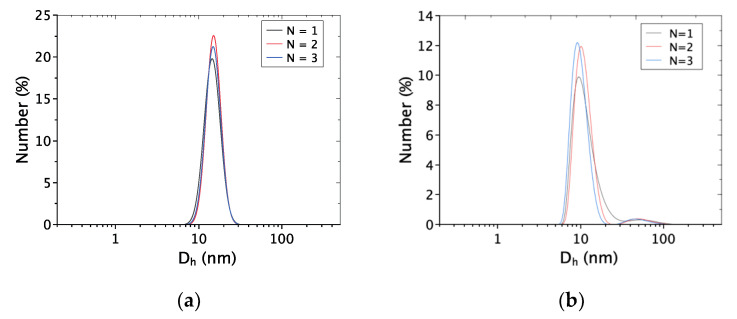
Dynamic Light Scattering (DLS) measurements for three independent experiments of (**a**) the AuNPs and (**b**) the AgNPs-aqueous solution (102.4 µM of metal). Number versus hydrodynamic diameter (*D*_h_) in nanometers.

The hydrodynamic diameters of nanoparticles suspended in water obtained by Dynamic light scattering (DLS) measurements are reported in Figure 2. For AuNPs (Figure 2a), only one peak is observed, whereas the AgNPs signal exhibits two peaks (Figure 2b).

The average hydrodynamic diameter for AuNPs is 13.5 ± 3.0 nm (Figure 2a). For AgNPs, the main population has an average size of 13.6 ± 3.2 nm (Figure 2b). By considering TEM observations, the minority population with a size ranging from 20 to 100 nm could be attributed either to the aggregation of AgNPs in solution (weak interaction) or to the presence of bigger AgNPs not observed by TEM. The absence of such big AgNPs during TEM observations may be related to the difference between TEM sampling (few drops) and the one used for DLS measurement (≥1 mL).

#### 2.1.3. UV-Visible Spectroscopy

Plasmonic nanoparticles, such as AuNPs and AgNPs, can also be characterized by UV-visible spectroscopy. One advantage is the sampling used, which is comparable to the one used for DLS measurements.

**Figure 3:**
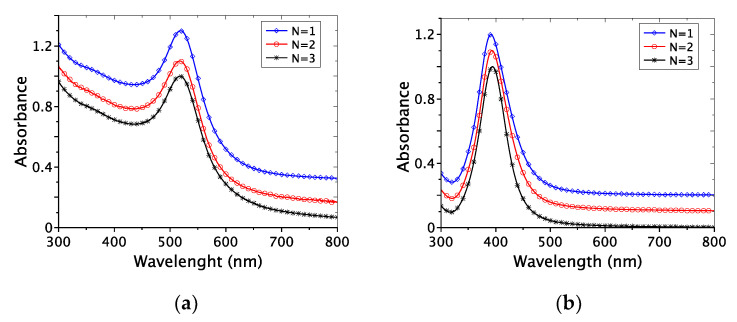
UV-visible spectra of (**a**) AuNPs and (**b**) AgNPs in water (for three independent experiments: *N* = 1 to 3). Stacked curves for more clarity.

Figure 3 shows the UV-vis spectra for three independent experiments for AuNPs and AgNPs. It highlights the repeatability of the procedure used for NPs preparation, with a maximum wavelength at 518 ± 1 nm and 391 ± 2 nm, for AuNPs and AgNPs (Figure 3a,b), respectively. In the case of AuNPs, the fitting programs distributed by V. Amendola have been used to estimate the average size of the nanoparticles [17]. The average size obtained for the AuNPs samples is 11.6 ± 3 nm, which is in good agreement with TEM observations and DLS measurements.

Following Mie’s theory [18], the AgNPs’ average size does not exceed 20 nm (λmax = 391 ± 2 nm). Furthermore, the full width at half maximum of 57 ± 4 nm is characteristic of low size distribution. Moreover, there is no peak for a wavelength higher than 450–500 nm. These observations allow us to attribute the signal observed in DLS around 20–100 nm to the aggregation of AgNPs in solution (Figure 2b).

#### 2.1.4. Surface Accessibility and Reactivity

The NPs antibacterial activity may imply NPs/bacteria interactions. Moreover, bacteria cell wall surfaces possess a negative zeta potential (i.e., −10 mV for *S. aureus* ATCC 12600 and −8 mV at 37 °C for *E. coli* ATCC 25922) [19,20], as well as the AuNPs and AgNPs (−43 mV and −40 mV, respectively). Hence, the catalytic reduction of 4-nitrophenol was chosen as a model reaction to study the NPs’ surface accessibility and reactivity, involving reagent and reactants negatively charged.

**Figure 4:**
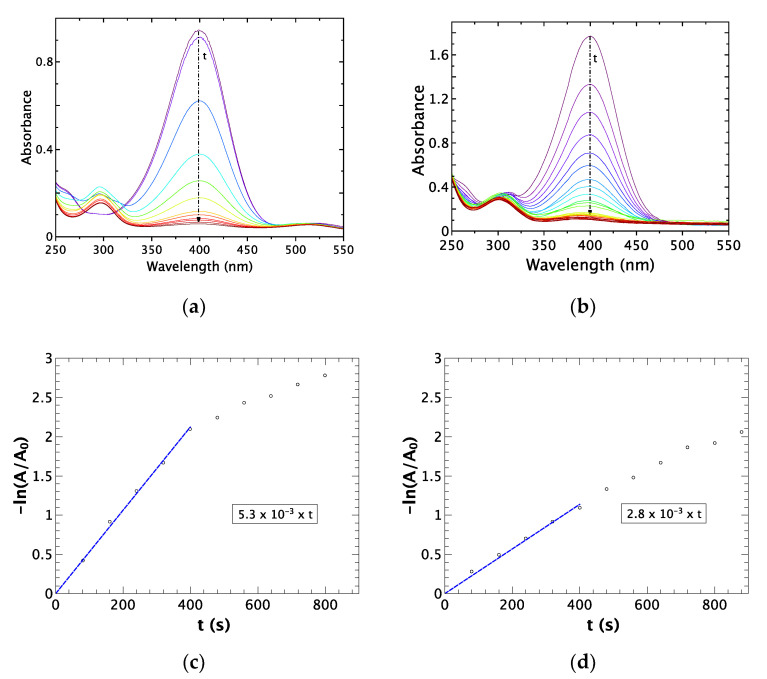
UV-vis spectra of 4-nitrophenol reduction as function of time and chemical reaction kinetics determination, respectively for (**a**,**c**) AuNPs and (**b**,**d**) AgNPs (pH = 10; T = 35 °C; 5 µg of metal; 10 μL of 4-nitrophenol at 1 × 10^−2^ µmol/L and 50 μL of NaBH_4_ at 0.4 µmol/L).

The results obtained for the 4-nitrophenol catalytic reduction by NaBH_4_ (35 °C and pH = 10) are shown in Figure 4 (a and b for AuNPs and AgNPs, respectively). In both cases, as t increases, the absorption peak around 400 nm decreases, while the one around 300 nm increases. These absorption variations are linked to the consumption of the 4-nitrophenol and the formation of the 4-aminophenol, respectively. As this reaction is from the first order, the corresponding reaction rate constants (k) can be determined from the slope of the linear plot Figure 4c,d): 5.3 × 10^−3^ s^−1^ for AuNPs, and 2.8 × 10^−3^ s^−1^ for AgNPs. As these k values are in good agreement with previously reported ones [21,22,23], we can conclude that both samples AuNPs and AgNPs exhibit good surface accessibility.

### 2.2. Antibacterial Activity

Antibacterial activity evaluation was performed by the broth microdilution method using Cation-Adjusted Mueller Hinton Broth (CA-MHB) as a liquid culture medium.

However, before facing bacteria, the stability of the NPs in bacterial growth conditions (i.e., 24 h at 35 °C in the culture medium, which is a complex mixture) has to be checked. Indeed, proteins and cations, which are part of the culture medium, could interact with the NPs surface. Such kind of interactions could lead to either NPs surface destabilization/passivation or NPs dissolution. Hence, without stability checking, it would be challenging to attribute activity to metallic NPs.

#### 2.2.1. Nanoparticles Stability in the Culture Medium

**Figure 5:**
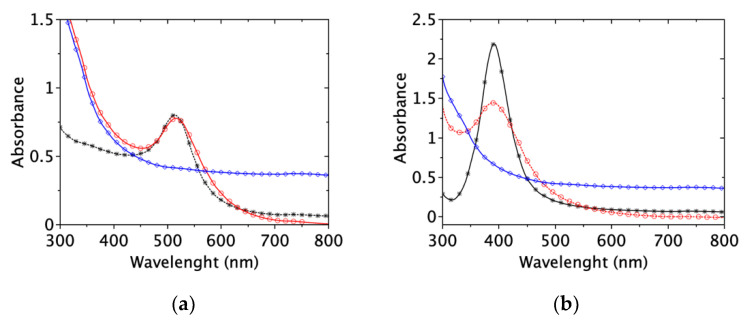
UV-visible spectra of 256 µmol/L of (**a**) AuNPs and (**b**) AgNPs in (*) water, in (○) CA-MHB, and in (◊) CA-MHB after 24 h at 35 °C.

As the Surface Plasmon Resonance phenomenon is sensitive to NPs size and surface state, 256 µmol/L of NPs in water and then in CA-MHB (based on metal content) were analyzed by UV-vis spectroscopy (Figure 5). Replacing water with CA-MHB (black stars and red circles on Figure 5, respectively) leads to a slight shifting of the maximum absorbance: From 519 to 520 nm for AuNPs, and from 391 to 390 nm for AgNPs. The elevated absorbance observed in the UV region corresponds to CA-MHB’s absorbance for both kinds of nanoparticles. After 24 h at 35 °C (blue rhombus in Figure 5), the characteristic absorbance peaks corresponding to AuNPs and AgNPs are no more observable.

According to Mie’s theory, the observed absorbance signal modifications can be attributed to the change of the NPs surface, which could be induced by (i) an interaction between the NPs surface and compounds from the bacterial culture medium [24], (ii) the NPs aggregation/agglomeration or (iii) a change of NPs morphology.

**Figure 6:**
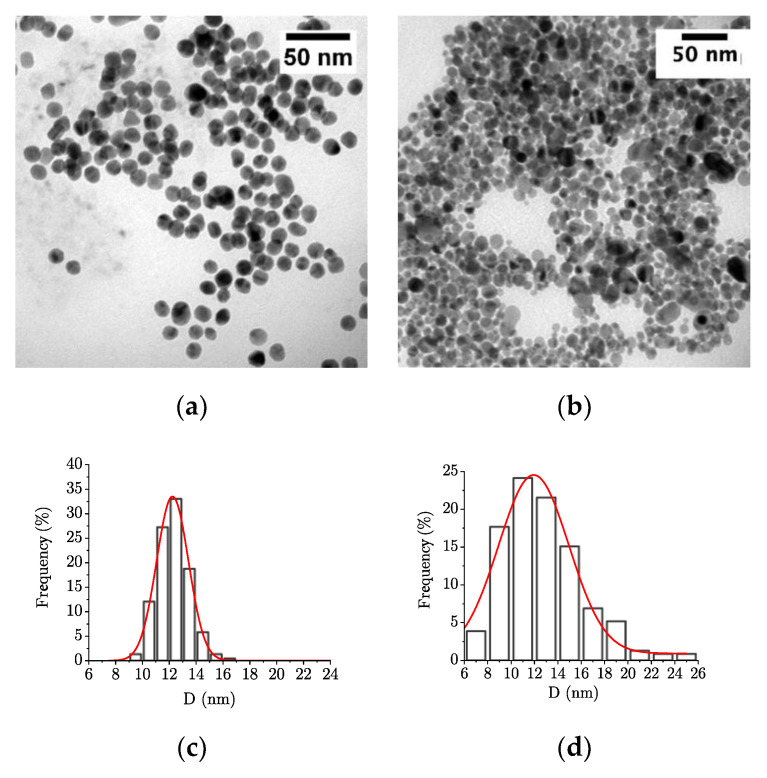
TEM micrographs and size distributions of (**a**,**c**) AuNPs and (**b**,**d**) AgNPs in CA-MHB.

To conclude about these modifications’ origin, the NPs samples have been observed by TEM after the following treatment 24 h at 35 °C in CA-MHB (Figure 6). AuNPs are isolated spheroidal nanoparticles, whereas spheroidal AgNPs appear a little more agglomerated (Figure 6a,b). The observed nanoparticles’ size distributions highlight mean sizes of 12.2 nm (σ = 1.2) and 11.9 nm (σ = 3.0) for AuNPs and AgNPs, respectively. A statistical test to compare them to the mean sizes obtained for NPs in water (i.e., 12.1 nm, σ = 1.1; and 12.2 nm, σ = 2.7) allows concluding that the mean sizes are identical with a confidence level of 95%. Consequently, neither the mean size of the NPs nor their morphology is altered by the conditions used for bacterial growth. It is worth noticing that due to the complex broth composition, DLS analysis did not provide interpretable data.

#### 2.2.2. Nanoparticles Antibacterial Activity Evaluation

NPs and bacteria (*S. aureus* ATCC 29213 or *E. coli* ATCC 25922) are incubated in CA-MHB. The inoculum concentration in bacteria is around 5.10^5^ CFU/mL. A range of NP concentration from 256 to 1 µmol/L is obtained by two-fold dilutions. After incubation for 24 h at 35 °C, the absorbance at 540 nm, which depends on the bacteria concentration, allows the evaluation of the bacteria growth.

**Figure 7:**
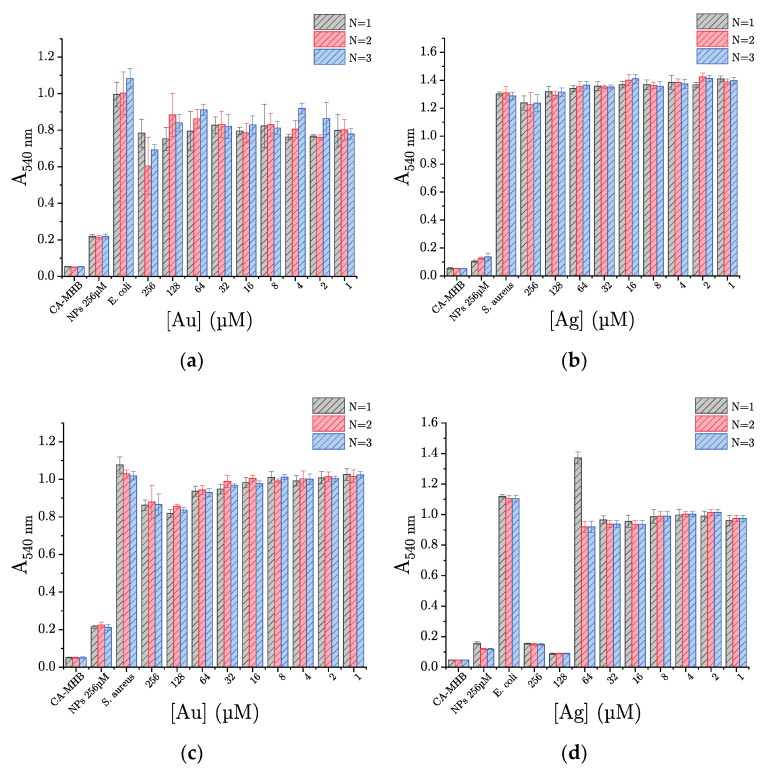
AgNPs and AuNPs antibacterial activity evaluation against (**a**,**b**) *Staphylococcus aureus* ATCC 29213 and (**c**,**d**) *Escherichia coli* ATCC 25922. “CA-MHB”: Culture medium control (i.e., culture medium free of bacteria and nanoparticles); “NPs 256 µM”: Nanoparticles control (i.e., bacteria-free culture medium, but with nanoparticles at 256 µmol/L); “*S. aureus* or *E. coli*”: Bacterial growth control (i.e., growing bacteria free of nanoparticles).

Figure 7 shows the obtained results for AuNPs (Figure 7a) and AgNPs (Figure 7b). The values obtained for the culture medium control (i.e., “CA-MHB”) and the nanoparticle control (i.e., “NPs 256 µmol/L”), with absorbances of 0.05 ± 0.01 and 0.20 ± 0.10, respectively, compared to that obtained for the bacterial growth control, allow concluding that there is no measurable bacterial contamination of both samples. The controls of bacteria growth, with an absorbance equal or higher than 1.00, allow checking the growth of *S. aureus* and *E. coli* in the absence of NPs.

Whatever the AuNPs concentrations tested against either *S. aureus* or *E. coli*, all measured absorbances are greater than those of NPs and culture medium controls (Figure 7a,c). Thus, in our experimental conditions (concentration ranging from 256 to 1 µmol/L), no minimum inhibitory concentration (MIC) is reached. However, if we look closely and compare the bacterial growth control absorbances to the value obtained for the highest AuNPs concentration tested (i.e., 256 µmol/L), a significant decrease is observed. Thus, at the concentration of 256 µmol/L, AuNPs at least partially inhibit bacterial growth, with an efficiency of around 12–14% and 20% against *S. aureus* and *E. coli*, respectively.

**Figure 8:**
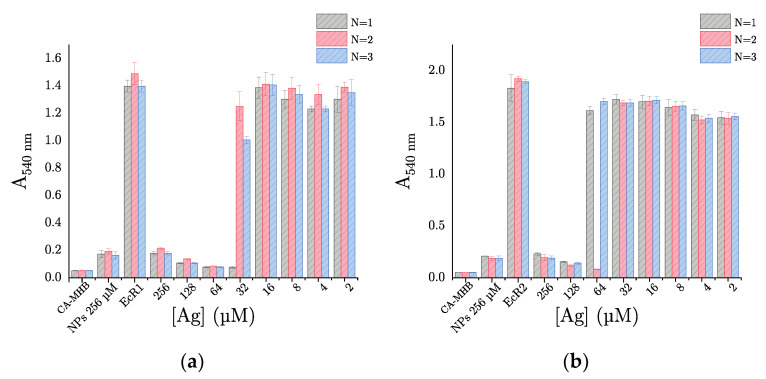
Evaluation of antibacterial activity by broth microdilution method results for AgNPs facing (**a**) EcR1 (ABC 23) penicillinase and (**b**) EcR2 (ABC 24) cephalosporinase. “CA-MHB”: culture medium control (i.e., culture medium free of bacteria and nanoparticles); “NPs 256 µM”: Nanoparticles control (i.e., bacteria-free culture medium, but with nanoparticles at 256 µmol/L); ”EcR1 or EcR2”: Bacterial growth control (i.e., growing bacteria free of nanoparticles).

In the case of AgNPs, the antibacterial activity appears to depend on the tested bacteria. When *S. aureus* is incubated with AgNPs, the measured absorbances are equivalent to that of the bacterial growth control, regardless of the concentration tested (Figure 7b). So, in our experimental conditions, AgNPs demonstrate no antibacterial activity against *S. aureus*. In contrast, AgNPs exhibit an evident antibacterial activity against *E. coli*. Indeed, for concentration in metal higher or equal to 128 µmol/L, an absorbance lower than 0.2, equivalent to the NPs control, is measured (Figure 7d). Thus, the bacterial growth is totally inhibited at these concentrations, and obviously, we can determine a MIC = 128 µmol of metal per liter for AgNPs against *E. coli*. Besides, given our encouraging results on a wild-type strain of *E. coli* (i.e., with no acquired antibiotic resistance), we decided to evaluate the antibacterial activity of AgNPs against two clinical isolates of *E. coli* resistant to β-lactams: EcR1 (i.e., penicillinase) and EcR2 (i.e., cephalosporinase overproduction). Unexpectedly, our results show that AgNPs inhibit the bacterial growth of both human clinical strains in a comparable way, with a MIC of 64 and 128 µmol of metal per liter, against EcR1 and EcR2, respectively (Figure 8a,b). AgNPs would therefore have the same antibacterial activity against *Escherichia coli*, whether it is resistant or not to antibiotics (i.e., β-lactams).

## 3. Discussion

The investigation of MNPs (i.e., metallic aggregates) antibacterial activity implies to consider four key points. Firstly, it is necessary to adequately characterize the MNPs size and size distribution and the sample homogeneity (i.e., a significant sampling). Secondly, the stabilizer potential antibacterial activity should be known. Thirdly, the MNPs surface accessibility and their stability in culture medium have to be established. Fourthly, referenced bacteria strains or well-characterized clinical isolates (i.e., strains with their antibiograms) should be used for antibacterial activity evaluation. At this price, it is possible to correctly assess the antibacterial activity and attribute it only to the MNPs (i.e., “naked” metallic aggregates). Following this approach, we studied the MNPs (with M = Au or Ag) antibacterial activity against *E. coli* and *S. aureus*, two of the most frequent bacterial species encountered in human infections [25]. The stabilizer (i.e., citrate) do not exhibit antibacterial activity in the concentration range used [26]. The MNPs sample characterizations were consistent in that they consist of spherical 12 nm gold and silver NPs with a narrow size distribution. Moreover, both their size and morphology were not altered when introduced into the bacterial culture medium. According to the literature, four to seven different mechanisms of action for MNPs as an antibacterial agent are described [27,28,29,30,31,32,33,34]. For example, Lee and Jun described four main routes of antibacterial mechanism of AgNPs, namely (i) adhesion to the cell membrane, (ii) penetration onside the cell, (iii) ROS generation and cellular toxicity, and (iv) genotoxicity. In a more recent review, Joshi et al., listed seven different mechanisms of action: (i) Disruption of cell membrane, (ii) destabilization and disruption of membrane proteins, (iii) destabilization and disruption of cytoplasmic proteins, (iv) inactivation of enzymes and metabolic interference, (v) generation of ROS, (vi) damage to DNA and ribosomal assembly, and (vii) impairment in transmembrane electron transport system [34]. It is worth noticing they can be coupled with each other. Even if the precise antibacterial mechanism of MNPs is still not elucidated, all of the suspected mechanisms imply a direct interaction between metallic atoms and bacteria. However, 12 nm citrate-capped MNPs (M = Ag, Au) possess a negative zeta potential, as for *E. coli* and *S. aureus*; so, as high positive or negative zeta potential values induce high repulsive forces [35], interactions between MNPs and bacteria can be lowered. Using a model catalytic reaction, such as the nitrophenol reduction by NaBH_4_ in the presence of MNPs, can remove such uncertainty. Indeed, as NaBH_4_ (itself negatively charge) solution is stable at basic pH values, the nitrophenol with its pKa of 7.15 at 25 °C [36] will be present as nitrophenolate anions. So, performing this catalytic reaction at basic pH involves reactant and reagent negatively charged. The catalytic results obtained highlight the zeta potentials of both 12 nm citrate-capped MNPs (M = Ag, Au), and the bacteria membrane will not limit their interaction.

Then, the antibacterial activity of AuNPs and AgNPs was evaluated against two bacteria: *E. coli* (ATCC 25922) and *S. aureus* (ATCC 29213). Doing so, we were unable to determine a MIC for AuNPs on the two bacteria. At best, we were able to demonstrate a partial inhibition of bacterial growth at the highest concentration tested (i.e., 256 µmol/L). On the other hand, even though AgNPs demonstrated no antibacterial activity against *S. aureus*, we have clearly determined a MIC = 128 µmol/L for the same AgNPs, under the same experimental conditions, against *E. coli*. Our results clearly show an antibacterial activity (even low) of AuNPs against *E. coli* ATCC 25922 and *S. aureus* ATCC 25923, which strongly differs from recent studies that failed to report any significant antibacterial activity of AuNPs against the same bacterial strains [37,38]. *A contrario*, for AgNPs, our results seem to be consistent with other studies which found that citrate-capped silver NPs showed a higher activity against *E. coli* than *S. aureus* [39,40,41,42]. For example, minimal bactericidal concentrations (MBC) of 9.94 µg/mL (i.e., ≈92 µM of metal) and 19.88 µg/mL (i.e., ≈184 µM) were obtained by C. Quintero-Quiroz et al., for AgNPs (5–50 nm) against *E. coli* ATCC 25922 and *S. aureus* ATCC 29213, respectively [40]. However, with AgNPs of 95.5 nm in diameter (large size distribution) facing the same strains, M. Zarei et al., reported no antibacterial activity against *E. coli*, while they determined a MIC of 5 µg/mL (i.e., ≈46 µM of metal) against *S. aureus* [43]. Moreover, 12.9 ± 4.5 nm AgNPs, prepared from silver nitrate, sodium citrate and ascorbic acid, tested against *E. coli* (MG 1655) and *S. aureus* (ATCC 6538) led to the determination of MBC of 0.14 mg/mL (i.e., 1.3 µM) and 0.35 mg/mL (3.2 µM) respectively [41]; whereas AgNPs ranging from 1 to 20 nm facing *E. coli* (ATCC 25922) and *S. aureus* (ATCC 12600) resulted in MIC of 0.049 mg/mL (i.e., ≈454 µM) and 0.391 mg/mL (i.e., 3625 µM) [42]. The differences observed with our results can be attributed to the AgNPs large size distribution [40,43], the presence of an antibacterial agent in the NPs preparation procedure [41], or the selection of different bacteria strains and concentration range (more specifically when *S. aureus* is under consideration) [41,42,43]. Nevertheless, it is noteworthy that MIC values obtained for our AgNPs against *E. coli* are in good agreement with those from other studies [40,44].

At this stage, and based on our results, we can make two hypotheses: Either AgNPs or AuNPs do not have exactly the same mechanism of action (since the AgNPs completely inhibit the growth of *E. coli* while the AuNPs only partially inhibit the growth of the two bacterial species); or the antibacterial action mechanism of the AgNPs and AuNPs depends on the bacterium tested, and more specifically depends on the structure and composition of the bacterial wall (since AgNPs do not inhibit the growth of *S. aureus* at all, whereas we determined a MIC for these same nanoparticles on *E. coli*). Indeed, several studies demonstrated that AuNPs and AgNPs interacted electrostatically with cell wall lipopolysaccharides (in Gram-negative bacteria) and teichoic and lipoteichoic acids (in Gram-positive bacteria) [45,46,47,48,49,50,51]. Moreover, it has been postulated that according to Derjaguin–Landau–Verwey–Overbeek (DLVO) theory, the electrostatic repulsive forces and van der Waals attractive forces are involved in the interaction between nanoparticles and bacterial cells in an aqueous suspension [46]. But, several authors suggested that the “strength” of electrostatic interactions between MNPs and bacteria (which could correspond to first step of the antibacterial mechanism of action) were under the dependence of the polysaccharides cell wall composition, structure, density…, which vary between different bacterial strains and therefore could explain the difference of the antibacterial activity of MNPs in function of the tested bacteria and even the opposite results obtained [34].

Besides, as AMR is a major public health issue, we wanted to test the antibacterial activity of AgNPs against antibiotic-resistant human clinical isolates of *E. coli*: EcR1 (penicillinase) and EcR2 (cephalosporinase overproduction). These two resistance mechanisms are among the most frequently found in *E. coli* [25]. Surprisingly, we have also shown that AgNPs completely inhibit the growth of these two clinical isolates (i.e., EcR1 and EcR2), with MIC values identical to those obtained for the wild strain (i.e., *E. coli* ATCC 25922). Therefore, it would seem that the antibacterial mechanism of action of AgNPs is not counteracted by mechanisms of resistance to β-lactams, which inhibit the synthesis of peptidoglycan (i.e., major and essential constituent of the bacterial cell wall).

The absence of size and shape evolution of the AgNPs sample in CA-MHB indicates that it should be lite enough not to be observed if dissolution. Additionally, as 2.5 equivalent NaBH_4_ versus AgNO_3_ and AgNPs washing cycle, the antibacterial activity cannot be attributed to free Ag^+^ ions present in colloidal solution. However, after the AgNPs/bacteria interaction or internalization, few Ag^+^ ions should be released from the NPs and improve their antibacterial activity.

## 4. Materials and Methods

### 4.1. Materials

Chloroauric acid (HAuCl_4_ ·3 H_2_O) was obtained from Alfa-Aesar, Karlsruhe, Germany. Silver nitrate (AgNO_3_), sodium citrate (Na_3_C_6_H_5_O_7_), sodium borohydride (NaBH_4_), and absolute ethanol (CH_3_CH_2_OH) were purchased from Sigma-Aldrich, Steinheim, Germany. The standard silver TraceCERT^®^ and gold TraceCERT^®^ came from the same supplier.

For the antimicrobial test, Cation-Adjusted Mueller–Hinton Broth (CA-MHB) from BBL™ (Batch 7291628) was prepared following the manufacturer’s instructions. Bacterial strains used, *Staphylococcus aureus* (ATCC 29213) and *Escherichia coli* (ATCC 25922), came from the American Type Culture Collection (ATCC); while EcR1 (ABC 23) and EcR2 (ABC 24) were *Escherichia coli* clinical isolates resistant to β-lactams (penicillinase and cephalosporinase overproduction, respectively) and came from ABC^®^ Platform Bugs Bank. All strains were grown in CA-MHB.

Ultrapure water (15 MΩ cm filtered at 0.22 μm) was used in all procedures.

### 4.2. Citrate-Capped Nanoparticles Synthesis

#### 4.2.1. Gold Nanoparticles

Sodium citrate was used to reduce chloroauric acid and NPs stabilization according to Turkevich procedure [16]. Briefly, chloroauric acid (HAuCl_4_, 3H_2_O: 1 × 10^−4^ mol) in water (100 cm^3^) was brought to a boil without refrigerant. A solution containing 5 molar equivalents of sodium citrate (i.e., 5 × 10^−4^ mol) in water (5 cm^3^) was heated and added to the auric solution. The heating was switched off after 5 min. After return to room temperature, the nanoparticles were centrifuged for 30 min at 5 °C at 8500× *g*. The supernatant was removed. Further purification of the sample was made 3 times as follows the addition of water (10 cm^3^), centrifugation (30 min, 5 °C, 8500× *g*) and removal of the supernatant. Finally, the colloidal solution was diluted to obtain the desired concentration.

#### 4.2.2. Silver Nanoparticles

Silver nanoparticles (AgNPs) were prepared by reducing silver nitrate by sodium borohydride in the presence of sodium citrate. Silver nitrate (AgNO_3_: 2 × 10^−5^ mol) in water (79.5 cm^3^) was prepared at room temperature. A solution containing 1 molar equivalent of sodium citrate (i.e., 2 × 10^−5^ mol) in water (0.5 cm^3^) was added to this solution of silver nitrate. Then, 0.5 mL of a sodium borohydride solution (NaBH_4_: 5 × 10^−5^ mol) was added dropwise (60 mL/h) to the silver solution. After one hour under agitation, the solution was centrifuged for 60 min at 8500× *g* at 5 °C. The supernatant was removed. Further purification of the sample was made 3 times as follows the addition of water (10 cm^3^), centrifugation (60 min, 5 °C, 8500× *g*) and removal of the supernatant. Finally, the colloidal solution was diluted to obtain the desired concentration.

### 4.3. Characterizations Methods

#### 4.3.1. UV-Visible Absorbance Spectroscopy

To measured surface plasmonic resonance and check the repeatability of synthesis, aliquots of colloidal solution were analyzed by measuring the UV-Visible spectrum at a resolution of 1 nm at 20 °C using UV-Visible spectroscopy (Perkin Elmer, Shelton, CT, USA, series LAMBA 1050).

#### 4.3.2. Transmission Electron Microscopy

The morphology and size of nanoparticles were determined using transmission electron microscopy (Philips, Tokyo, Japan, CM200). Colloidal solutions were diluted in absolute ethanol, then few drops of ethanol colloidal solution were loaded onto a carbon-coated copper grid. After evaporating the excess solvent, nanoparticles were visualized using TEM, which was operated at a 20 kV accelerating voltage. Images treatment was realized using ImageJ software (v1.51m9, NIH, Bethesda, MD, USA) [52].

#### 4.3.3. Dynamic Light Scattering

Hydrodynamic sizes were determined using Multi-Angle Dynamic Light Scattering with Malvern Zetasizer Nano ZS by the combination of the signal at 13°, 90°, and 173° from source (λ = 633 nm) in plastic cuvettes.

#### 4.3.4. Inductively Coupled Plasma Spectroscopy

Determination of metal (i.e., Au or Ag) concentration in aqueous colloid solution was performed using Inductively Coupled Plasma with detector by Absorption Emission Spectrometer (ICP-AES) with ICP Ultima (Jobin-Yvon Horiba, Kyoto, Japan). The concentration was determined according to TraceCERT^®^ standard. The samples were prepared by digestion of nanoparticles in acid solution, aqua regia for AuNPs, and nitric acid for AgNPs.

### 4.4. Surface Accessibility

The surface accessibility was checked by nanoparticles’ ability to catalyze the reduction reaction of 4-nitrophenol in 4-aminophenol in an aqueous solution at pH = 10. In a quartz cuvette, 40 μL of nanoparticles at 1 mmol/L of metal, 10 μL of 4-nitrophenol at 1 × 10^−2^ mol/L, and 50 μL of NaBH_4_ at 0.4 mol/L at pH 10 were stirring. The reaction was monitored by recording UV-visible spectra between 250 and 800 nm every 10 s for 2 min, then each 30 s afterward.

### 4.5. Stability Study

Stability in CA-MHB was evaluated using %absorbance measurements, between 250 and 800 nm, for nanoparticles at 256 µmol/L of metal at t = 0 and t = 24 h at 35 °C. TEM images were recorded with these samples, and statistical tests have been performed to check the nanoparticle size evolution. For this purpose, with a population of 500 NPs, the bilateral student test and Fisher-Snedecor test were used to compare the mean diameters and the variances, respectively.

### 4.6. Antimicrobial Evaluation

Nanoparticles’ antibacterial activities were determined using methods based on the broth microdilution method [53]. Briefly, twofold serial dilutions of drugs were prepared in CA-MHB in 96-well microtiter plates (Greiner, Bernolsheim, France, 650161), starting from a stock aqueous solution of 1024 µmol/L (number of mol of metal atom/L considered) to obtain a final concentration range from 256 to 1 µmol/L^1^. Then, normed inoculum prepared from bacteria in the stationary phase, at 5.10^5^ to 5.10^6^ colony forming unit (CFU) by mL, was put in each well. Three controls were realized: Culture medium alone, nanoparticles in culture medium at 256 µmol/L, and bacteria without nanoparticles. After incubation for 24 h at 35 °C under agitation at 150 RPM, bacterial growth was evaluated with an ELISA plate reader (read at 540 nm, Multiskan EX, Thermo Electron Corporation, Saint-Herblain, France). All shown results are expressed as the means ± standard deviation of 8 wells at the same concentration of 96-wells microtiter plates. The three independent determinations are presented on the same graphic.

## 5. Conclusions

The purpose of the present study was to determine whether MNPs (i.e., metallic aggregates) can be considered new potential antibacterial agents. To provide some element of the answer, adequately characterized 12 nm citrate-capped gold and silver nanoparticles have been tested against *S. aureus* (ATCC 29213) and *E. coli* (ATCC 25922). In this study, we evidenced that both AuNPs and AgNPs are stable in CA-MHB at 35 °C over 24 h. Besides, the results clearly indicate that AuNPs exhibit only light antibacterial activity against *S. aureus* and *E. coli*. In contrast, AgNPs possess high antibacterial activity only against *E. coli*. We assume that the difference in the cell-wall structure of these bacteria (e.g., presence of an external membrane in Gram-negative bacteria, like *E. coli*) explains the difference in some bacteria sensitivity (depending on the composition/structure of their cell wall) to AgNPs. Moreover, AgNPs antibacterial activity appears to be maintained against antibiotic-resistant *E. coli* strains, presenting either a penicillinase or a cephalosporinase-overproduction. This study suggests that among MNPs (with M = Au or Ag), only AgNPs can be considered a new potential antibacterial agent. However, the scope of this study was limited in terms of NPs size and bacteria number. Hence, it would be interesting to assess the effects of NPs size and higher NPs concentrations on the antibacterial activity and the antibacterial spectra of the AgNPs, in particular other MDR-bacteria.

## Data Availability

The data that support the findings of this study are available from the corresponding author upon reasonable request.
